# Alteration of E2F2 Expression in Governing Endothelial Cell Senescence

**DOI:** 10.3390/genes13091522

**Published:** 2022-08-24

**Authors:** Hongfei Liu, Liping Chen, Wanli Xiao, Jiankun Liu, Changkun Long, Wenxing Zhan, Cui Cui, Lin Yang, Shenghan Chen

**Affiliations:** 1 Vascular Function Laboratory, Human Aging Research Institute and School of Life Science, Jiangxi Key Laboratory of Human Aging, Nanchang University, Nanchang 330031, China; 2 Metabolic Control and Aging, Human Aging Research Institute and School of Life Science, Jiangxi Key Laboratory of Human Aging, Nanchang University, Nanchang 330031, China; 3 Aging and Vascular Diseases, Human Aging Research Institute and School of Life Science, Jiangxi Key Laboratory of Human Aging, Nanchang University, Nanchang 330031, China; 4 Department of Ophthalmology, Handan Central Hospital, Handan 056001, China; 5 Department of Nephrology, Taikang Southwestern Medical Center, Chengdu 610213, China

**Keywords:** cell cycle, E2F2, endothelial cell, proliferation, senescence

## Abstract

Endothelial cell senescence has a vital implication for vascular dysfunction, leading to age-related cardiovascular disease, especially hypertension and atherosclerosis. E2F transcription factor 2 (E2F2) plays a critical role in cell proliferation, differentiation, and DNA damage response. Up to date, no study has ever connected E2F2 to vascular endothelial cell senescence. Here, we demonstrate that E2F2 is involved in endothelial cellular senescence. We found that E2F2 expression is decreased during the replicative senescence of human umbilical vein endothelial cells (HUVECs) and the aortas of aged mice. The knockdown of *E2F2* in young HUVECs induces premature senescence characterized by an increase in senescence-associated β-galactosidase (SA-β-gal) activity, a reduction in phosphorylated endothelial nitric oxide synthase (p-eNOS) and sirtuin 1 (SIRT1), and the upregulation of senescence-associated secretory phenotype (SASP) IL-6 and IL-8. The lack of E2F2 promoted cell cycle arrest, DNA damage, and cell proliferation inhibition. Conversely, *E2F2* overexpression reversed the senescence phenotype and enhanced the cellular function in the senescent cells. Furthermore, *E2F2* deficiency downregulated downstream target genes including CNNA2, CDK1, and FOXM1, and overexpression restored the expression of these genes. Our findings demonstrate that E2F2 plays an indispensable role in endothelial cell senescence.

## 1. Introduction

Vascular endothelial cells compose the inner layer of blood vessels, which is fundamental for homeostasis and is extremely vulnerable to hemodynamic stress [1,2]. During the aging process, endothelial cells undergo senescence, decreased proliferation, and a loss of vascular repair capability, thereby leading to a decline in endothelial-dependent vasomotor function [3]. The impairment of aging vessels is attributed to DNA damage, telomere attrition, and cellular senescence [4,5,6]. The phenotype of vascular aging is illustrated by enlarged vascular lumen, a thickened intima-media layer, decreased small vessel density, increased collagen, and decreased elastin deposition [7]. It is known that vascular aging is associated with an increased incidence of cardiovascular disease, especially hypertension and atherosclerosis, and an advanced age accelerates vascular endothelial dysfunction [8,9,10,11]. Endothelial senescence is considered as a significant mediator for vascular aging and age-related disease. The senescent cells exhibit common features including elevated SA-β-galactosidase (SA-β-gal) activity, the increased expression of cyclin-dependent kinase (CDK) inhibitors (P21, P16), augmented senescence-associated secretory phenotype (SASP), declined endothelial nitric oxide synthase (eNOS) activity, and decreased sirtuin 1 (SIRT1) expression [3,7,12,13]. Numerous genes related to cell cycle regulation, oxidative stress, energy metabolism, inflammation, and neurohormonal control are implicated in vascular endothelial cell senescence [7].

E2F transcription factor 2 (E2F2), one of the E2F family members, is identified as the cell cycle activator, which is critical for proliferation, differentiation, DNA damage response, and cell death [14,15,16]. E2F2 in cell cycle regulation exhibits cell-type-specific, such as positive regulation for cells such as fibroblasts, hepatocyte and hematopoietic progenitors, cardiomyocytes, and thymic epithelial cells and negative regulation for T lymphocytes and chondrocytes [17,18,19,20,21]. The inhibition of E2F2 activity reduces cell proliferation in cancer development [22]. E2F2 also plays an important role in regulating the expression of PCSK6, which modulates cardiomyocyte senescence [23,24]. In vivo, E2F2 knockout mice display impaired endothelial function, increased vessel contraction, and high blood pressure [25]. Up to date, however, there is no study connecting E2F2 to vascular endothelial cell senescence.

We, in this study, characterized the role of E2F2 in endothelial cell senescence and explored the possible downstream molecular signaling by the endothelial replicative senescence model. We demonstrate that E2F2 is involved in vascular endothelial senescence and cellular function during the aging process.

## 2. Materials and Methods

### 2.1. Cell Culture

Human umbilical vein endothelial cells (HUVECs) were extracted from an umbilical cord and cultured in Endothelial Cell Medium (ScienCell, San Diego, CA, USA). All cells were maintained at an incubator with 5% CO_2_ at 37 °C.

### 2.2. Mice

Male C57BL/6 mice were acquired from the Model Animal Research Center of Nanchang University and kept on 12 h light/dark cycles with free access to food and water in ventilated cages. All experimental procedures were performed in accordance with the Guide for the Care and Use of Laboratory Animals from the Human Aging Research Institute of Nanchang University.

### 2.3. Quantitative Real-Time PCR (qPCR)

The total RNA from the cells was isolated with TRIzol reagent (Thermo Fisher Scientific, Waltham, MA, USA) and reverse-transcribed by a commercial kit (Zomanbio, Beijing, China). qPCR with M5 HiPer Realtime PCR Super Mix (Mei5bio, Beijing, China) was carried out using qTOWER3 G Real Time PCR Systems (Analytik Jena, Jena, Germany). The primer sets for the human genes are detailed below: 

E2F2, 5′-CGC ATC TAT GAC ATC ACC AA-3′ and 5′-CAA ACA TTC CCC TGC CTA C-3′; 

CDKN2B, 5′-GAG GAG AAC AAG GGC ATG-3′ and 5′- CTC CCG AAA CGG TTG ACT-3′; 

E2F1, 5′-AGA CCG TAG GTG GGA TCA G-3′ and 5′-TAT GGT GGC AGA GTC AGT GG-3′; 

CCNA2, 5′- TGT CAC CGT TCC TCC TTG-3′ and 5′-GGC ATC TTC ACG CTC TAT-3′; 

CCNB1, 5′-GGA AGA GCA AGC AGT CAG-3′ and 5′-CTA GCC AGT CAA TTA GGA TG-3′; 

CCNB2, 5′-CCC TTG CCA CTA CAC TTC-3′ and 5′-AGA GTC AGC TCC ATC AAA TAC-3′; 

CDK1, 5′-GAT GTG CTT ATG CAG GAT T-3′ and 5′-TGT ACT GAC CAG GAG GGA-3′; 

CDC25A, 5′-GAG GAT GAT GGC TTC GTG-3′ and 5′-ATC GGT TGT CAA GGT TTG TAG T-3′; 

FOXM1, 5′-TGG AGC AGC GAC AGG TTA-3′ and 5′-TGC TGT TGA TGG CGA ATT-3′; 

MYBL2, 5′-CGG AGC CCC ATC AAG AAA-3′ and 5′-GCA GTT GTC GGC AAG GAT-3′; 

ACTIN, 5′-AGA GGG AAA TCGT GCG TGA C-3′ and 5′-CAA GAA GGA AGG CTG GAA AA-3′. 

The Ct values were achieved based on the amplification curve. The relative gene expression was estimated by the 2^−ΔΔCt^ method.

### 2.4. Transfection of siRNA and Adenovirus DNA

The HUVECs were cultured to 70% confluency and then transfected with 50 nM siRNA with Lipofectamine 2000 (Invitrogen, Carlsbad, CA, USA) or 2.5 × 10^5^ PFU/mL adenovirus expressing human *E2F2* (ADV-*E2F2*) (Obio, Shanghai, China) for 48 h according to the reagent instruction manual. The sequences of scramble siRNA and siRNAs targeting the human *E2F2* gene (si*E2F2*-1 and si*E2F2*-2) were as follows. Scramble siRNA: 5′-UUC UCC GAA CGU GUC ACG UTT-3′; si*E2F2*-1: 5′-GGA CAA CCU GCA GGA UAU AUT T-3′; and si*E2F2*-2: 5′-CCG UGC UGU UGG CAA CUU UTT T-3′. 

### 2.5. SA-β-gal Staining

The detection of SA-β-gal activity was carried out by the Senescence β-Galactosidase Staining Kit (Beyotime, Shanghai, China). The HUVECs were fixed by a fixative solution at room temperature for 10 min. Then, the cells were incubated with an X-gal staining solution at 37 °C for 16 h. The percentage of blue-stained cells was calculated under a microscope.

### 2.6. Immunofluorescent Staining

The fixation of cells or tissue sections was performed with 4% (vol/vol) paraformaldehyde for 10 min at room temperature and followed by permeabilization in PBS containing 0.25% Triton X-100 for 10 min. The samples were blocked and then incubated with a primary antibody including anti-E2F2 (1:100, Abcam, Cambridge, UK), anti-phosphorylated histone H2AX (γH2AX) (1:150, CST, Danvers, MA, USA), and anti-CD31 (1:100, R&D, Minneapolis, MN, USA) at 4 °C overnight. Afterwards, Cy3-conjugated secondary antibody (1:200, Biolegend, San Diego, CA, USA) or Cy5-conjugated secondary antibody (1:200, Jackson ImmunoResearch, West Grove, PA, USA) was added to the samples and incubated in the dark for 1 h at room temperature. The nuclear counterstaining was carried out by Hoechst33342 (Beyotime, Shanghai, China). Photos were taken with a confocal microscope (Carl Zeiss, Oberkochen, BW, Germany).

### 2.7. Protein Extraction and Western Blotting 

The cells were lysed by RIPA buffer (Solarbio, Beijing, China) for protein extraction. The concentration of protein was determined with a BCA Protein Assay Kit (Beyotime, Shanghai, China). Western blot analysis was performed as described previously [24]. The primary and secondary antibodies used in this study include E2F2 (1:3000, Abcam, Cambridge, UK), P21 (1:3000, Proteintech, Wuhan, Hubei, China), p-eNOS (1:2000, Abcam, Cambridge, UK), SRIT1 (1:2000, Thermo Fisher Scientific, Waltham, MA, USA), GAPDH (1:10,000, Proteintech, Wuhan, Hubei, China), HRP-conjugated goat anti-rabbit IgG (1:10,000, Proteintech, Wuhan, Hubei, China), and HRP-conjugated goat anti-mouse IgG (1:10,000, Proteintech, Wuhan, Hubei, China). An analysis of the band intensities was conducted with ImageJ (NIH, Bethesda, MD, USA) by normalizing to GAPDH.

### 2.8. Cell Cycle Analysis

Cell cycle analysis was performed with the Cell Cycle and Apoptosis Analysis kit (Beyotime, Shanghai, China). The HUVECs were harvested and washed once with PBS. Then, the cells were resuspended with 70% ethanol at 4 °C overnight. After the ethanol was removed, the cells were washed twice with PBS. The cells were incubated with a propidium iodide (PI) solution containing RNase for 30 min. The percentage of cells in each phase of the cell cycle was detected by FACSverse (BD Biosciences, San Jose, CA, USA).

### 2.9. EdU Cell Proliferation Assay 

The proliferation of HUVECs was detected by the EdU-488 Cell Proliferation Detection Kit (Beyotime, Shanghai, China). The HUVECs were incubated with the EdU solution for 2 h in an incubator with 5% CO_2_ at 37 °C. The cells were fixed and stained with Azide-fluor 488. The nuclear counterstaining was carried out by Hoechst33342. Photos were taken with ImageXpress Confocal HT.ai (Molecular Devices, San Jose, CA, USA).

### 2.10. ELISA

The concentration of interleukin 6 (IL6) and interleukin 8 (IL8) from the conditioned medium was measured by the homo IL6 and IL8 ELISA kit (Proteintech, Wuhan, Hubei, China). The medium was collected and centrifuged at 1000× *g* for 5 min to remove cell debris. After adding the stop solution, the optical density values were determined using SpectraMax i3x (Molecular Devices, San Jose, CA, USA) at 450 nm.

### 2.11. RNA-Sequencing Analysis

GSE172094 (www.ncbi.nlm.nih.gov/geo/query/acc.cgi (accessed on 14 October 2021)) and GSE155680 (www.ncbi.nlm.nih.gov/geo/query/acc.cgi (accessed on 11 January 2022)), from publicly available Gene Expression Omnibus (GEO) datasets, were used for bioinformatics analysis. Differential expression analysis was performed by DESeq2 (v1.4.5). Log2 foldchange ≥ 1 and qvalue < 0.05 are defined as the thresholds for differential expression. The heatmap was built up with a pheatmap (v1.0.12) base on the gene expression. Kyoto Encyclopedia of Genes and Genomes (KEGG) and Gene Ontology (GO) enrichment analyses were conducted by the WEB-based GEne SeT AnaLysis Toolkit (www.webgestalt.org, accessed on 14 October 2021). Gene interaction analysis was performed on the String website (cn.string-db.org, accessed on 14 October 2021).

### 2.12. Statistical Analysis

The statistical analysis was executed using GraphPad PRISM version 8.4.2 (La Jolla, CA, USA). All data are displayed as the mean ± SD. One-way ANOVA with Dunnett’s multiple comparison was applied for comparing three or more groups, and a two-tailed Student’s *t*-test was applied for comparing two groups. Two-way ANOVA was used for multiple comparisons with two variables. A *p* value < 0.05 is statistically significant.

## 3. Results

### 3.1. E2F2 Expression Is Decreased in the Senescent Endothelial Cells and the Aorta of Aged Mice

**Figure 1:**
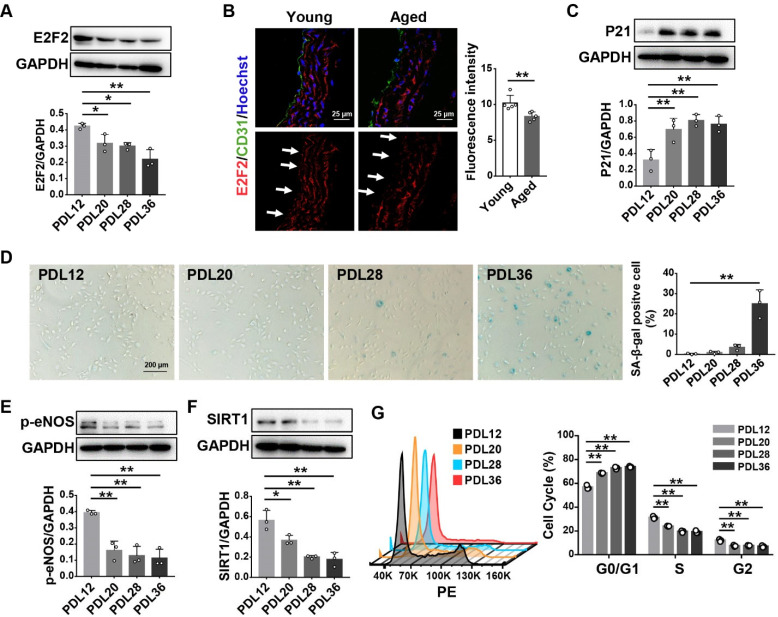
Decreased E2F2 expression in senescent HUVECs and in the aortas of aged mice. (**A**) E2F2 expression in HUVECs with different PDLs by Western blotting. Protein levels, normalized to GAPDH, were estimated by densitometry. Statistical analysis was performed with one-way ANOVA. (**B**) Immunofluorescent staining for E2F2 (red), CD31 (green), and nuclei (blue) in frozen aorta sections from young mice (3 months, *n* = 5) and aged mice (15 months, *n* = 5). Scale bar: 25 µm. Arrows indicate endothelium. Statistical comparison of mean fluorescent intensity was performed by Student’s *t* test. (**C**) Protein expression of P21 in HUVECs with different PDLs. (**D**) SA-β-gal staining (blue) for HUVECs with different PDLs. Scale bar: 200 µm. One-way ANOVA for a comparison of the percentage of SA-β-gal-positive cells. Protein expression of p-eNOS (**E**) and SIRT1 (**F**) in HUVECs with different PDLs. (**G**) Cell cycle analysis for HUVECs with different PDLs. Statistical analysis was performed with two-way ANOVA. Values are mean ± S.D. * *p* < 0.05, ** *p* < 0.01.

To investigate whether E2F2 expression changes with aging, we carried out the experiments using a replicative senescence model at different population doubling levels (PDL), as well as with aged mice. PDL12 was defined as young cells, and PDL20, 28, and 36 were defined as senescent cells [26]. We found that the E2F2 protein expression level was lower in the HUVECs at PDL20-36 than that of PDL12 (Figure 1A). A decrease in the E2F2 level was also observed in the aorta of aged mice (15 months) compared to that of young mice (3 months) (Figure 1B). We verified the HUVECs with CD31, which is a specific marker for endothelial cells [27]. Furthermore, the senescence marker P21 was increased in PDL20-36 cells in comparison with that in PDL12 cells (Figure 1C). SA-β-gal activity, a biomarker for the identification of senescent cells [28], was elevated and characterized by more blue-stained cells at PDL20-36 (Figure 1D). The senescence-related genes p-eNOS and SIRT1 showed lower levels in PDL20-36 HUVECs than in PDL12 cells (Figure 1E,F). The cell cycle progression was suppressed at the G0/G1 phase in the senescent cells (Figure 1G). All the data indicate that E2F2 expression is decreased during cellular senescence and aging.

### 3.2. E2F2-Knockdown Induces Endothelial Cell Senescence

**Figure 2:**
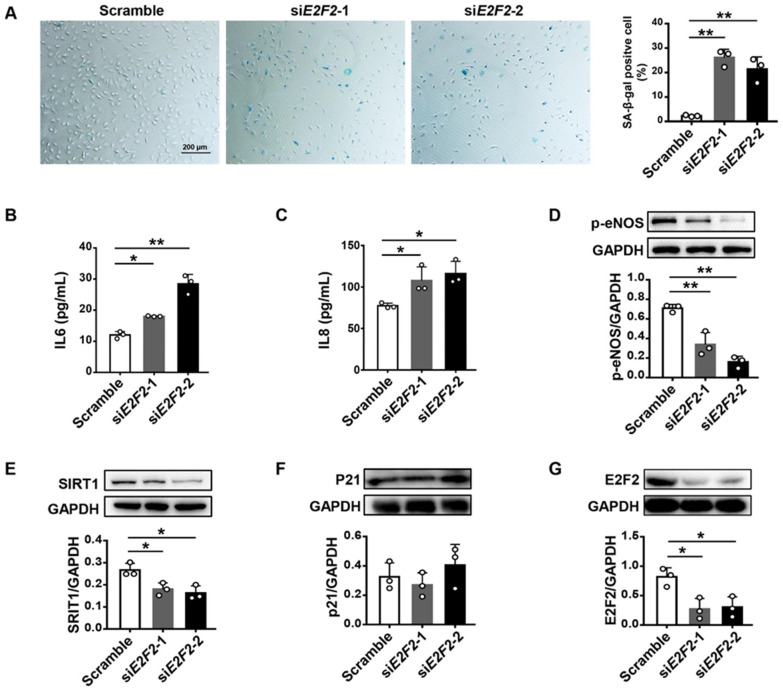
Increased endothelial cell senescence in *E2F2*-knockdown HUVECs (PDL12). (**A**) SA-β-gal staining (blue) for HUVECs transfected with siRNAs (si*E2F2* and si*E2F2*). Scale bar: 200 µm. One-way ANOVA for a comparison of the percentage of SA-β-gal-positive cells. (**B**,**C**) ELISA for IL6 and IL8 levels in *E2F2*-knockdown HUVECs. One-way ANOVA for statistical analysis. Protein expression of p-eNOS (**D**), SIRT1 (**E**), P21 (**F**), and E2F2 (**G**) in HUVECs transfected with two sets of si*E2F2*. Protein levels, normalized to GAPDH, were estimated by densitometry. Statistical analysis was performed with one-way ANOVA. Values are mean ± S.D. * *p* < 0.05, ** *p* < 0.01.

In light of these findings, we inspected whether a deficiency of E2F2 results in endothelial cell senescence. We knocked down the E2F2 gene with two sets of siRNA (si*E2F2*-1 and si*E2F2*-2) in young HUVECs at PDL12. SA-β-gal activity and SASP IL-6 and IL-8 from a conditioned medium were found to be significantly increased in *E2F2*-knockdown cells compared to those of the scramble (Figure 2A–C). The protein levels of p-eNOS and SIRT1 were decreased when knocking down *E2F2* (Figure 2D,E). However, there was no difference observed in P21 expression between cells with or without the treatment of siRNA (Figure 2F). Western analysis verified a higher efficiency of E2F2 knockdown in HUVECs (Figure 2G). These results suggest that a lack of E2F2 leads to endothelial cell senescence.

### 3.3. E2F2-Knockdown Impairs Endothelial Cellular Function

**Figure 3:**
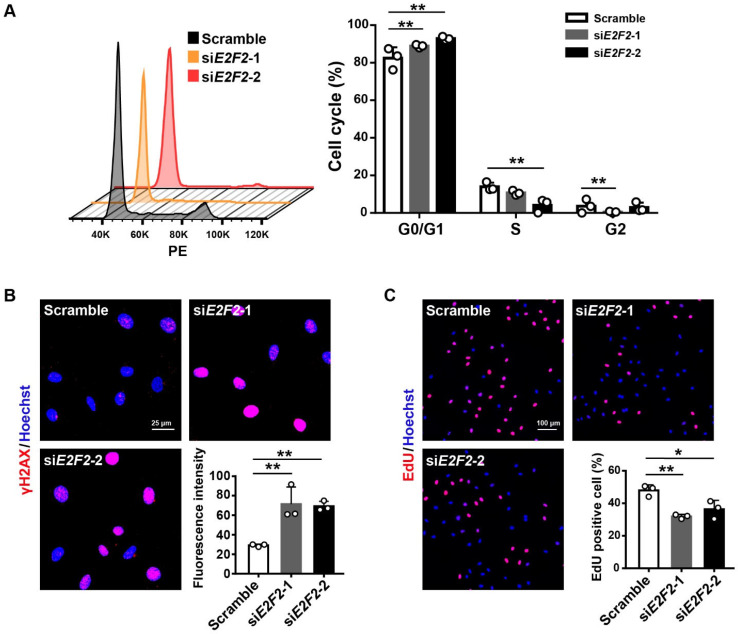
Impaired endothelial cell function in *E2F2*-knockdown HUVECs (PDL12). (**A**) Cell cycle analysis for HUVECs transfected with si*E2F2*s. Statistical analysis by two-way ANOVA. (**B**) Immunofluorescent staining for the level of γH2AX in *E2F2*-knockdown HUVECs. Scale bar: 25 µm. Statistical comparison of mean fluorescent intensity using one-way ANOVA. (**C**) EdU for the cell proliferation of *E2F2*-knockdown HUVECs. Scale bar: 100 µm. One-way ANOVA for a comparison of the percentage of EdU-positive cells. Data are means ± S.D. * *p* < 0.05, ** *p* < 0.01.

We next examined the function of HUVECs at PDL12 upon *E2F2* deficiency. Flow cytometry analysis showed that the cell cycle was arrested at the G0/G1 phase in the cells transfected with si*E2F2*s as compared to the scramble (Figure 3A). We further evaluated the DNA damage level by detecting the intensity of γH2AX, a DNA damage marker [29], in *E2F2*-knockdown HUVECs and found an increased γH2AX level of cells (Figure 3B). In addition, the EdU incorporation assay showed that cell proliferation was inhibited by the knockdown of *E2F2* (Figure 3C). These experiments demonstrate that a lack of E2F2 promotes endothelial cellular dysfunction. 

### 3.4. Overexpression of E2F2 Attenuates the Senescence Phenotype of Endothelial Cells 

**Figure 4:**
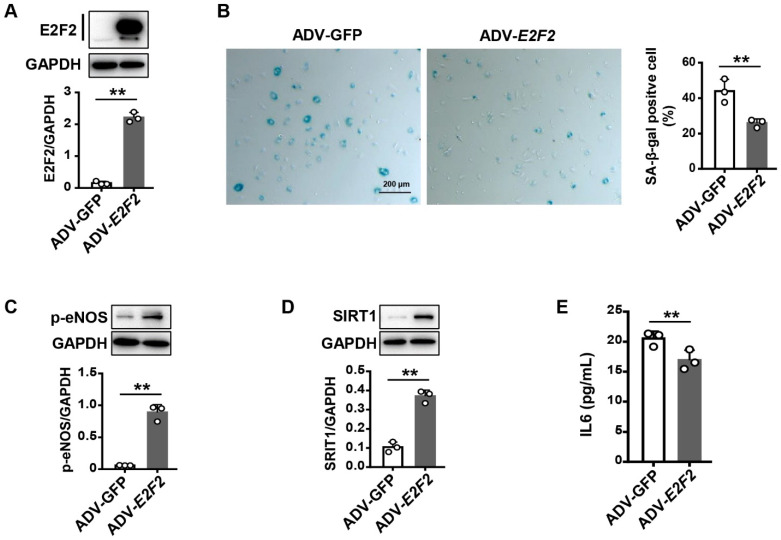
Rescue of endothelial cell senescence in *E2F2*-overexpressed HUVECs (PDL36). (**A**) Western blot analysis for HUVECs transfected with ADV-*E2F2*. ADV-GFP as a control. (**B**) SA-β-gal staining (blue) for HUVECs transfected with ADV-*E2F2*. Scale bar: 200 µm. Protein expression of p-eNOS (**C**) and SIRT1 (**D**) in HUVECs transfected with ADV-*E2F2*. (**E**) ELISA for the IL6 level in the conditioned medium of *E2F2*-overexpressed cells. Student’s *t* test for statistical analysis. Values are mean ± S.D. ** *p* < 0.01.

To verify the role of E2F2 in endothelial cell senescence, we overexpressed *E2F2* by ADV-*E2F2* in the senescent HUVECs (PDL36). The efficiency of overexpression was evaluated by Western analysis (Figure 4A). The percentage of SA-β-gal positive cells was remarkably reduced in PDL36 cells transfected with ADV-*E2F2* compared to ADV-GFP control cells (Figure 4B). Likewise, lower expression levels of p-eNOS and SIRT1 were recovered in the senescent cells treated with ADV-*E2F2* (Figure 4C,D). Moreover, the IL6 level was decreased in the conditioned medium of senescent HUVECs with *E2F2* overexpression (Figure 4E). The results indicate that the overexpression of *E2F2* reverses the senescence phenotype in endothelial cells.

### 3.5. E2F2 Overexpression Reverses Endothelial Dysfunction in Senescent Endothelial Cells

**Figure 5:**
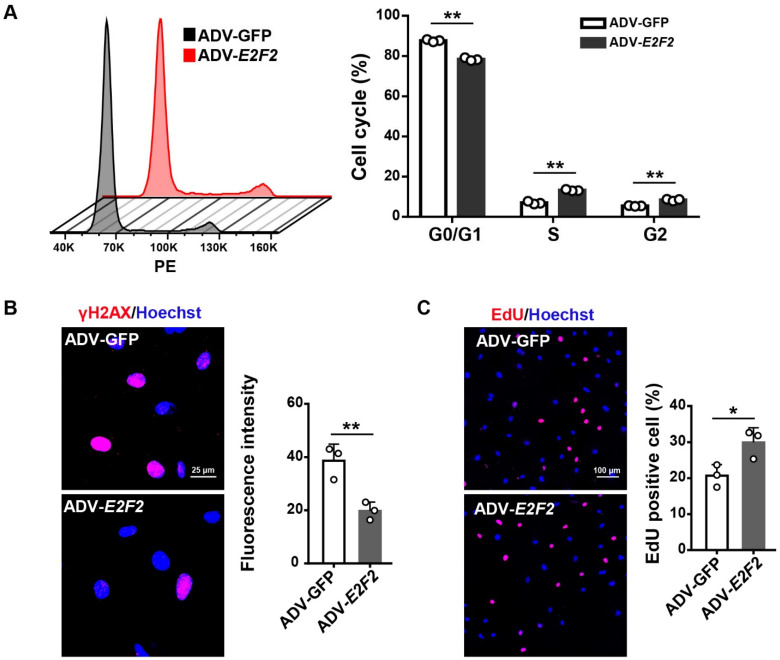
Restored endothelial cell function in *E2F2*-overexpressed HUVECs (PDL36). (**A**) Cell cycle analysis for HUVECs transfected with ADV-*E2F2*. Statistical analysis by two-way ANOVA. (**B**) Immunofluorescent staining for the level of γH2AX in *E2F2*-overexpressed HUVECs. Scale bar: 25 µm. Student’s *t* test for the statistical comparison of mean fluorescent intensity. (**C**) EdU for cell proliferation in *E2F2*-overexpressed HUVECs. Scale bar: 100 µm. Student’s *t* test for a comparison of the percentage of EdU-positive cells. Data are means ± S.D. * *p* < 0.05, ** *p* < 0.01.

We further tested whether *E2F2* overexpression improves cellular dysfunction in senescent HUVECs. By flow cytometry, we found that G0/G1 phase accumulation in the senescent cells was attenuated after overexpressing *E2F2* (Figure 5A). DNA damage detected by γH2AX immunocytochemistry was lessened in the senescent cells transfected with ADV-*E2F2* compared to that with ADV-GFP (Figure 5B). Similarly, the cell proliferation was restored in senescent HUVECs with the transfection of ADV-*E2F2* (Figure 5C). These findings suggest that the overexpression of *E2F2* reinstates cellular function in senescent endothelial cells. 

### 3.6. Differentially Expressed Genes (DEGs) Are Identified in E2F2-knockdown and Senescent Endothelial Cells

**Figure 6:**
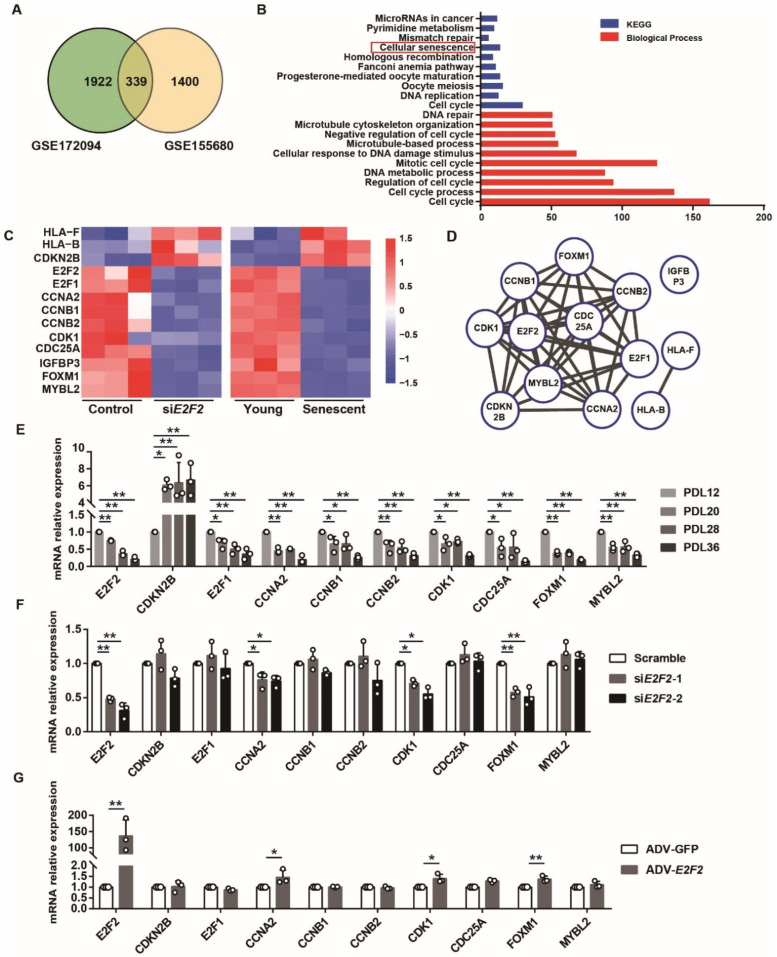
Identifying differentially expressed genes (DEGs) in *E2F2*-knockdown and senescent endothelial cells. (**A**) Venn diagram for the intersection of DEGs in *E2F2*-knockdown and senescent cells. GSE172094: siRNA-mediated E2F2 silencing in growth-stimulated prostate cancer cells; GSE155680: young and senescent endothelial cells; log2 Fold Change ≥ 0.5, qvalue < 0.05. (**B**) KEGG and GO enrichment analysis for DEGs in signaling pathways. (**C**) A heatmap of 13 DEGs associated with cellular senescence. The color intensity denotes the relative gene expression, upregulation (red), and downregulation (blue). (**D**) A gene interaction network for targeted genes related to cellular senescence by String. qPCR for mRNA expression of 10 DEGs in HUVECs with different PDLs (**E**), *E2F2*-knockdown HUVECs (PDL12) (**F**) and *E2F2*-overexpressed HUVECs (PDL36) (**G**). One-way ANOVA for statistical comparison. Values are mean ± S.D. * *p* < 0.05, ** *p* < 0.01.

Our data illustrated that E2F2 plays a part in endothelial senescence. Furthermore, we attempted to identify the molecular pathways downstream from E2F2. We analyzed two datasets retrieved from the publicly available GEO. One is GSE172094 (siRNA-mediated E2F2 silencing in growth-stimulated LNCaP prostate cancer cells), and the other is GSE155680 (young and senescent HUVECs). Overall, 339 DEGs were identified as overlapping between *E2F2*-silencing LNCaP prostate cancer cells and the senescent HUVECs (Figure 6A). KEGG and GO enrichment analysis showed that these DEGs were clustered in various pathways including cellular senescence, cell cycle, and DNA repair (Figure 6B), in which a total of 13 genes were screened out in the pathway of cellular senescence (Figure 6C). Among them, 10 genes were found to interact with each other by network analysis (Figure 6D). qPCR validated that the mRNA levels of these 10 genes were decreased in the replicative senescence model (Figure 6E). After knocking down *E2F2*, CCNA2 (cyclin A), CDK1 (cyclin dependent kinase 1), and FOXM1 (forkhead box M1) were significantly downregulated in the PDL12 HUVECs (Figure 6F). Notably, in the senescent cells, only these three genes repressed by E2F2 silencing were rescued by the overexpression of E2F2 (Figure 6G). All the results confirmed that E2F2 modulates the expression of specific downstream genes related to cellular senescence and the cell cycle, suggesting the possible signaling of E2F2 in senescence.

## 4. Discussion

In the present study, we found that *E2F2* expression is decreased during the replicative senescence of endothelial cells as well as the natural aging of aortas. The knockdown of *E2F2* in endothelial cells induces premature senescence and cellular dysfunction in young HUVECs, while *E2F2* overexpression rescues this phenotype and enhances the cell function in the senescent cells. Additionally, *E2F2* deficiency downregulated target genes, and overexpression promotes the expression of these genes. Our findings demonstrate that *E2F2* plays an indispensable role in endothelial cell senescence.

The development of cellular senescence is crucial for endothelial dysfunction related to vascular aging. Endothelial cells experience replicative senescence, which is characterized by a declined proliferation capacity that can be assessed by PDL [7]. We introduced an endothelial replicative senescence model as an in vitro setting into our study, and this model is verified by the classic senescence indicators such as SA-β-gal, P21, cell cycle progression, and the senescence signatures eNOS and SIRT1 [12,30,31]

The fundamental feature of senescent cells is the persistent arrest of the cell cycle, leading to diminished proliferation [32,33]. Upon knocking down E2F2 in young HUVECs, we observed similar phenotypes to the replicative senescence, including elevated SA-β-gal activity, increased IL6 and IL8 levels, deceased p-eNOS and SIRT1 expression, and cell cycle arrest. Interestingly, the senescence marker P21 was not affected in *E2F2*-deficient cells. The available evidence shows that E2F2 is controlled by cyclin-dependent kinases and their inhibitors. However, E2F2 induces the expression of cell cycle protein such as CCNA2 but does not increase the P21 level [34,35]. The previous report indicates that P21 expression may not be necessary for human fibroblasts senescence [36]. Further study reveals that the senescence-associated phenotype and cell cycle arrest are not definitely tethered in senescent P21-deficeint fibroblasts; however, a low level of P21 is sufficient to facilitate the senescent cell cycle arrest [37]. It is found that neural stem cells treated with irradiation exhibit the senescence phenotype without the significant upregulation of P21 [38]. The senescence induced by interleukin-32θ shows a decreased level of E2F2 and unaltered P21 expression [39]. Thus, endothelial cell senescence induced by E2F2 deficiency may be irrespective of P21 expression. This implies a novel mechanism of E2F2 in modulating senescence—possibly P21, as upstream signaling impacting E2F2 [40]. Moreover, our study shows that the knockdown of E2F2 suppresses the cell cycle at the G0/G1 phase in young HUVECs, which is in agreement with a G1 arrest in senescent endothelial cells, thereby stopping the entrance into the S phase [28,35]. The overexpression of E2F2 in the senescent cells recaptures the function. 

The other hallmark of cellular senescence is excessive SASP secretion. Upon senescent cells buildup, they release plenty of SASP proteins to induce neighboring normal cell senescence and to promote cellular function deterioration, which exacerbates tissue aging. On the other hand, SASP can eliminate the senescent cells through the immune system and repair damaged tissues [41,42]. As a result, the SASP proinflammatory phenotype is presented in arteries with an advanced age [43]. The most consistently age-related biomarkers are IL6 and IL-8 [10,44]. Our data showed that IL6 and IL8 levels were increased in *E2F2*-deficient young cells and reversed in *E2F2*-overexpressed senescent cells. SASP production is also stimulated by a persistent DNA damage response [45]. Accumulative unrepaired DNA damage contributes to vascular aging [46]. DNA damage response and genomic instability are recognized as characteristics of cellular senescence and aging [4,47]. E2F2 has been reported to prevent DNA damage and maintain genomic stability in a number of cell types, including neuronal cells, embryonic fibroblasts, and T lymphocytes [48,49]. We detected that a lack of E2F2 resulted in more DNA damage in young HUVECs; in contrast, the overexpression of E2F2 counteracts increased DNA damage in the senescent cells. Such evidence highlights a role of E2F2 in endothelial cell senescence. 

It is well-documented that senescence changes gene expression in vascular endothelial cells. In this study, bioinformatics analysis identified three downstream target genes of E2F2 including CNNA2, CDK1, and FOXM1. We found that CNNA2, a member of the cyclin family, is expressed in young HUVECs and decreases with an increasing PDL, which is consistent with previous reports [35,50]. As in the case of replicative senescence, CNNA2 mRNA expression is inhibited in *E2F2*-knockdown cells. Conversely, the overexpression of *E2F2* enhances the CNNA2 level in the senescent cells. CDK1 belongs to the protein kinase superfamily, which presents a low expression and activity in the T lymphocytes of aged subjects [51]. There is evidence showing that CDK1 deficiency promotes cellular senescence [52]. The study also indicates that the knockdown of CCNA2 impacts CDK1 [52]. FOXM1 is a transcription activator which is involved in controlling cellular aging and improving the lifespan [53]. Hence, it is possible that E2F2 may drive vascular endothelial cell senescence from interacting with these senescence-associated target genes.

This study has some limitations. The cellular senescence induced by E2F2 seems to not be interrelated with alterations in the expression of p21. More studies are needed to address the underlying mechanism and define how E2F2 mediates endothelial senescence. The role of E2F2 in senescence at the cellular level requires validation in aged animal models and in aged blood vessels from humans. 

## Data Availability

The publicly available RNAseq datasets analyzed in this study are available at NCBI’s Gene Expression Omnibus (GSE172094 and GSE155680).
